# Synthesis 1-Acyl-3-(2'-aminophenyl) thioureas as Anti-Intestinal Nematode Prodrugs 

**DOI:** 10.3390/molecules15106941

**Published:** 2010-10-08

**Authors:** Li-Ping Duan, Jia Xue, Li-Li Xu, Hao-Bing Zhang

**Affiliations:** National Institute for Parasitic Diseases, Chinese Center for Disease Control and Prevention, Shanghai, 200025, China

**Keywords:** acyl, thiourea, deparasitization, * Nippostrongylus brazilliensis*

## Abstract

A series of 1-acyl-3-(2'-aminophenyl) thiourea derivatives were designed and synthesized. The structures of all the newly synthesized compounds were identified by IR, elemental analysis, ^1^H-NMR and ^13^C-NMR. Their anti-intestinal nematode activities against *Nippostrongylus brazilliensis* were evaluated in rats by an oral route. Among these compounds, at concentrations of 10 mg/kg of rat, compound (1-(2'-furanyl)acyl-3- (2'-aminophenyl) thiourea) (**5h)** produced the highest activity with 89.4% deparasitization. The present work suggests that 1-acyl-3-(2'-aminophenyl) thiourea derivatives may become useful lead compounds for anti-intestinal nematode treatment.

## 1. Introduction

Mebendazole and albendazole have been used against human and animal helminth parasites for more than two decades [1,2,3,4,5]. They are derived from benzimidazole which has a broad spectrum of activity and is used to treat nematode and trematode infections in domestic animals. The limited solubility of benzimidazoles may have a major influence on their absorption and clinical efficacy [6,7]. Furthermore, when used in lengthy therapies, they can produce side-effects, such as severe headaches, fever, fatigue, hair loss, and liver degeneration [8] and hence are not recommended for patients with hepatic problems. A way to overcome these problems is to use prodrugs [9,10,11], such as 4-amino-3-(3'-methoxycarbonyl-2'-thioureido)benzophenone [12]. It is a soluble prodrug, which is enzymatically cyclized to mebendazole *in vitro*. On the other hand, thiourea derivatives also exhibit potent antiviral, antibacterial and cytotoxic activities [13,14]. Several works demonstrate their activity against parasites such as *Plasmodium falciparum, Trichomonas vaginalis* and *Trypanosoma. cruzi* [15,16]. Based on these reports, we report herein the synthesis, characterization, and *in vitro* evaluation of anti-intestinal nematode activity of eight different novel thiourea derivatives bearing the *o*-aminobenzene moiety. 

## 2. Results and Discussion

### 2.1. Synthesis and Characterization of 1-Acyl-3-(2-aminophenyl) thiourea Derivatives ***5a-5h***

**Scheme 1:**
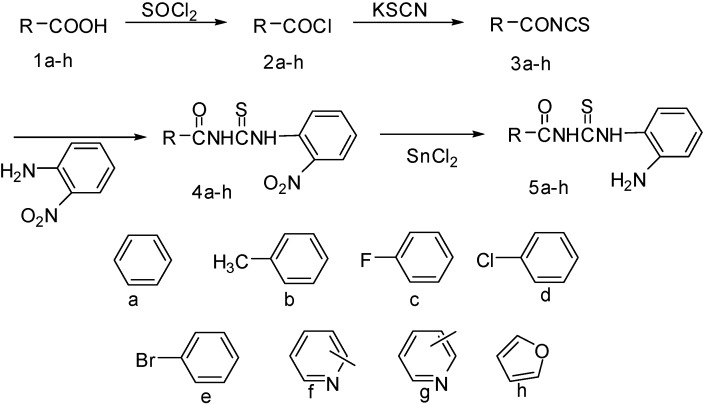
Synthesis of compounds **5a-5h**.

The synthetic route to the target compounds **5a-5h** is shown in Scheme 1. Firstly, acids **1a-1h** were acylated by SOCl_2_ followed by isothiocyanation and coupling reactions with 2-nitrobenzenamine to give 1-acyl-3-(2'-nitrophenyl) thioureas **4a-4h** in moderate yield. Then the title compounds **5a-5h** were successfully obtained in 50-60% overall yield using SnCl_2_ as reducing agent. Compounds **5a-5h** were characterized by ^1^H-NMR, ^13^C-NMR and elemental analysis. All results are in full agreement with the proposed structures. For example, the ^1^H-NMR spectrum of compound **5b** showed a singlet at 2.40 ppm (CH_3_), singlets at 12.9 and 11.6 ppm (NHCSNH) and a multiplet from δ = 7.91 to δ = 6.75 for aromatic hydrogens. Moreover, the ^13^C-NMR spectrum showed δ 29.6 (CH_3_), 154.19, 141.62, 133.95, 130.41, 125.70, 124.63, 122.27, 119.28, 115.78, 114.44, 113.15, 109.34 (benzene C), 165.1 (C=O), 180.2 (C=S), all consistent with its proposed structure. The elemental analyses results were in good agreement with those calculated for the suggested formulae. The melting points are sharp, indicating the purity of these compounds.

### 2.2. Anti-intestinal Nematode Activity

From Table 1, we can see that some of compounds showed significant anti-intestinal nematode activity in a two-day *in vivo* test in rats. At concentrations of 10 mg/kg of rat, compound **5h** produced the highest activity against *Nippostrongylus brazilliensis* with 89.4 % deparasitization. For anti-intestinal nematode activity, it appears that a variety of substituents can be introduced on the phenyl ring without significantly altering the activity relative to the unsubstituted phenyl analogue **5a**. For example, the substituted F, CH_3_, Cl, and Br acyl derivatives all have the almost same activity as **5a**. Moreover, the 3'-pyridylacyl derivative **5g** is slightly more active than the corresponding 2'-isomer **5f**. On the other hand, the structural variation between compounds **5b** and **5f** results in different activity. Compound **5a**, **5b**, **5c**, **5d** and **5e** contains benzene moieties, while **5f** and **5g** have a pyridyl group moiety. This pyridyl group appears to be particularly responsible for anti-intestinal nematode activity. Compounds **5f** and **5g**, which contain a pyridyl moiety, and **5h** that contains a furanyl moiety all seemed to be much more effective in terms of anti-intestinal nematode activity. Because compound **5h** displayed anti-intestinal nematode potency that is comparable to albendazole (10 mg/kg), further anti-intestinal nematode activity assay was carried out for compound **5h**. It was found that at concentrations of 18 mg/kg of rat, **5h** produced the highest activity against *Nippostrongylus brazilliensis* with 100%, effectiveness, which implies further possibilities for lead compound development.

**Table 1:** Results of the chemotherapeutic trials of thiourea derivatives bearing *O*-aminobenzene moieties in rats.

Number of worms recovered from rats (including worms in rats at *post mortem*)						
	Dose(mg/kg)	1	2	3	Average	Deparasitization(%)
**Control**	**0**	200	198	199	199	
**Albendazole**	10	0	0	0	0	100.00
**5a**	10	180	170	176	175	13.7
**5b**	10	178	180	178	178	10.5
**5c**	10	183	177	174	178	10.5
**5d**	10	188	183	184	185	7.0
**5e**	10	177	170	170	172	13.5
**5f**	10	109	110	108	109	45.2
**5g**	10	108	107	96	103	48.2
**5h**	10	22	23	19	21	89.4

## 3. Conclusions

In summary, various types of 1-acyl-3-(2-aminophenyl) thioureas were synthesized and their varying biological activities towards the *N. brazilliensis* was demonstrated. Among these compounds, **5h** produced the highest activity against *N. brazilliensis* with 89.4% deparasitization. The present work suggest that **5h** may be a useful lead compound for anti-intestinal nematode medicine development. Further studies of the structure-biology activity relationships around the designed compounds are underway.

## 4. Experimental

### 4.1. General

All the reagents and solvents were of the commercial quality and were used without purification. Elemental analysis was performed on a PE-2400 elemental analyzer, the C, H and N analysis were repeated twice. ^1^H-NMR and ^13^C-NMR spectra were obtained in DMSO-*d*_6_ with TMS as internal standard on a Bruker AM-400 spectrometer. Chemical shifts are reported as ppm. Melting points were determined by an X-6 micro-melting point apparatus and are uncorrected.

### 4.2. General Procedure for the Preparation of 1-Acyl-3-(2-aminophenyl)thioureas ***5a-5h***

According to our reported procedure [17] different acids **1a-1h** were treated with SOCl_2_ and KSCN, respectively, affording moderate yields of around 75% of the intermediates **3a-3h,** which were used directly without further purification. The subsequent nucleophilic reactions of **3a-3h** with 2-nitro-benzenamine led to the key intermediates **4a-4h**, respectively. Then reduction of **4a-4h** with SnCl_2_ in CH_3_COOH afforded the target compounds **5a-5h**, which were recrystallized twice from DMF/H_2_O.

*1-Phenylacyl-3-(2'-aminophenyl) thiourea* (**5a**): Yield 60%, mp 154~156^o^C. IR (KBr) : 3165, 1628, 1255 cm^−1^; ^1^H-NMR δ: 12.52 (s, 1H, NH), 11.44 (s, 1H, NH), 8.03-6.80 (m, 9H, Ar-H), 5.30 (s, 2H, Ar-NH); ^13^C-NMR δ: 180.34, 165.48, 154.39, 133.62, 132.95, 130.41, 130.10, 128.63, 128.27, 127.28, 125.78, 124.05, 119.32, 118.44; Anal. Calcd. for C_14_H_13_N_3_OS (271.2): C 61.97, H 4.83, N 15.49; found C 62.00, H 4.83, N 15.60.

*1-(4'-Methylphenyl)acyl-3-(2'-aminophenyl) thiourea* (**5b**): Yield 58%, mp 164~165^o^C. IR (KBr) : 3208, 1645, 1240 cm^−1^; ^1^H-NMR δ: 12.91 (s, 1H, NH), 11.64 (s, 1H, NH), 7.91-6.75 (m, 8H, Ar-H), 5.32 (s, 2H, Ar-NH), 2.40 (s, 3H, CH_3_); ^13^C-NMR δ: 180.24, 165.18, 154.19, 141.62, 133.95, 130.41, 125.70, 124.63, 122.27, 119.28, 115.78, 114.44, 113.15, 109.34, 29.64; Anal. calcd. for C_15_H_15_N_3_OS (285.1): C 63.13, H 5.30, N 14.73; found C 63.56, H 5.41, N 14.73.

*1-(4'-Fluorophenyl)acyl-3-(2'-aminophenyl) thiourea* (**5c**): Yield 53%, mp 173~174^o^C. IR (KBr): 3235, 1650, 1235 cm^−1^; ^1^H-NMR δ: 12.93 (s, 1H, NH), 11.67 (s, 1H, NH), 8.12-6.80 (m, 8H, Ar-H), 5.35 (s, 2H, Ar-NH); ^13^C-NMR δ: 180.27, 166.18, 165.27, 155.29, 140.18, 139.19, 134.62, 133.95, 126.41, 124.70, 123.68, 122.58, 121.20, 118.54; Anal. calcd. for C_14_H_12_FN_3_OS (289.2): C 58.12, H 4.18, N 14.52; found C 58.56, H 4.14, N 14.68.

*1-(4'-Chlorophenyl)acyl-3-(2'-aminophenyl) thiourea* (**5d**): Yield 58%, mp 182~184^o^C. IR (KBr): 3225, 1630, 1240 cm^−1^; ^1^H-NMR δ: 12.90 (s, 1H, NH), 11.34 (s, 1H, NH), 7.97-6.80 (m, 8H, Ar-H), 5.23(s, 2H, Ar-NH); ^13^C-NMR δ: 180.23, 165.19, 154.67, 141.13, 139.37, 136.89, 135.05, 130.78, 128.95, 126.73, 125.47, 121.40, 120.85, 119.44; Anal. calcd. for C_14_H_12_ClN_3_OS (305.7): C 54.99, H 3.96, N 13.74; found C 56.00, H 3.94, N 13.80.

*1-(4'-Bromophenyl)acyl-3-(2'-aminophenyl) thiourea*(**5e**): Yield 50%, mp 156~158^o^C. IR (KBr): 3230, 1680, 1245 cm^−1^; ^1^H-NMR δ: 12.70 (s, 1H, NH), 11.44(s, 1H, NH), 7.92-6.75 (m, 8H, Ar-H), 5.19(S, 2H, Ar-NH); ^13^C-NMR δ: 180.30, 165.21, 154.89, 141.19, 139.17, 136.69, 135.75, 130.80, 128.99, 126.80, 125.87, 122.67, 121.78, 120.56; Anal. calcd. for C_14_H_12_BrN_3_OS (350.2): C 48.01, H 3.45, N 12.00; found C 48.06, H 3.54, N 12.08.

*1-(2'-Pyridyl)acyl-3-(2'-aminophenyl) thiourea* (**5f**): Yield 78%, mp 180~181^o^C. IR (KBr): 3190, 1675, 1250 cm^−1^; ^1^H-NMR δ: 12.90 (s, 1H, NH), 11.68 (s, 1H, NH), 8.02-7.83 (m, 4H, Py-H), 7.03-6.75 (m, 4H, Ar-H), 5.20 (s, 2H, Ar-NH); ^13^C-NMR δ: 180.36, 166.90, 154.48, 151.23, 147.67, 137.50, 130.48, 126.76, 125.51, 124.56, 124.00, 119.96,114.90; Anal. calcd for C_13_H_12_N_4_OS (272.3): C 57.34, H 4.44, N 20.57; found C 57.36, H 4.44, N 20.68.

*1-(3'-Pyridyl)acyl-3-(2'-aminophenyl) thiourea* (**5g**): Yield 49%, mp 180~181^o^C. IR (KBr): 3215, 1665, 1242 cm^−1^; ^1^H-NMR δ: 12.91 (s, 1H, NH), 11.64 (s, 1H, NH), 8.02-7.83 (m, 4H, Py-H), 7.03-6.75 (m, 4H, Ar-H), 5.21 (s, 2H, Ar-NH); ^13^C-NMR δ: 180.36, 166.92, 154.48, 151.23, 147.57, 137.51, 130.48, 126.76, 125.55, 124.56, 124.02, 119.99, 114.90; Anal. calcd. for C_13_H_12_N_4_OS (272.3): C 57.34, H 4.44, N 20.57; found C 57.37, H 4.44, N 20.66.

*1-(2'-Furanyl)acyl-3-(2'-aminophenyl) thiourea* (**5h**): Yield 48 %, mp 121~122^o^C. IR (KBr): 3220, 1640, 1250 cm^−1^; ^1^H-NMR δ: 12.91 (s, 1H, NH), 11.64 (s, 1H, NH), 8.09-6.83 (m, 3H, furyl-H), 7.03-6.75 (m, 4H, Ar-H), 5.20 (s, 2H, Ar-NH); ^13^C-NMR δ: 180.30, 166.78, 154.45, 147.23, 143.65, 130.48, 125.53, 124.56, 119.63, 115.36, 109.71; Anal. calcd. for C_12_H_11_N_3_O_2_S (261.3): C 55.16, H 4.24, N 16.08; found C 55.20, H 4.24, N 16.09.

### 4.3. Biological Assays

All analogues were tested against *N. brazilliensis* to evaluate their anti-intestinal nematode activities using the screening method described by Cavier [18].. The compounds were dissolved in dimethyl formamide (DMF) and serially diluted with water containing Triton X-80 (0.1 mg/L) to get the required test concentrations. Each rat in the respective group received 10 mg/kg body weight using oral candle. These compounds were tested on ten groups of rats, each containing three rats. Evaluations were based on a percentage scale of 0-100, in which 100 was total kill and 0 was no activity. All results are shown in Table 1. The reference compound was albendazole, and water containing DMF (0.5 mg/L) and Triton X-80 (0.1 mg/L) was used as a negative control. The trials commenced on the 10th day after infecting each of 30 rats with 250 *N. brazilliensis* larvae. The percentage deparasitization was calculated using the following formula:

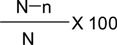

where N = average number of worms found in the control animals and n = average number of worms found in the groups of treated animals (including worms in rats at *post mortem*).
